# Dietetic Malnutrition in Infants and Its Treatment

**Published:** 1918-11

**Authors:** Frank Brundage

**Affiliations:** Asst. Pediatrist, University of Buffalo, N. Y.


### BUFFALO MEDICAL JOURNAL

Yearly Volume 74 NOVEMBER, 1918 Number 4

### ORIGINAL ARTICLES

The right is reserved to decline papers not dealing with practical medical and surgical subjects, and such as might offend or fail to interest readers. Contributors are solely responsible for opinions, methods of expression and revision of proof.

### Dietetic Malnutrition in Infants and Its Treatment.

FRANK BRUNDAGE, M. D., Asst. Pediatrist,

University of Buffalo.

In presenting for your consideration the subject of malnutrition I do not wish to disappoint your expectations for something new, either in regard to the diagnosis or treatment of the condition but rather to emphasize some important truths.

The recent contributions to our knowledge of this subject, particularly those of Finklestein and Meyer, have made a new classification of the disorders of digestion and malnutrition as described by the name of decomposition; but for the present, the term malnutrition will answer our purpose. Its occurrence is so common and its treatment so varied that a momentary review of the subject will, I hope, be productive of some good. Malnutrition is a secondary condition and not a disease, the degree of severity depending upon its cause and duration. It is characterized by a stationary or decreasing weight, a sensitive digestion, more or less anemia and an unbalanced nervous system. It occurs in both breast and bottle infants; in the former usually mild in degree but the latter running a more severe course. The primary causes are many, foremost among them being lues, tuberculosis, infectious diseases, and digestive disorders of the severe type. Of infantile malnutrition due to specific diseases it is not my intention to speak of at present but only of the malnutrition due entirely to faulty diet.

Owing to the prolonged use of proprietary foods or the unskilled use of cow's milk, by far the greater number of cases occur in the bottle fed. In the breast fed, malnutrition occurs in about half as many cases as in the bottle fed and is here due to a diminished quantity or quality of breast milk or a disproportion of its constituents. The matter of environment, too, is often a contributary factor yet in some of the worst cases, the environment may be all that could be desired but the degree of malnutrition is severe—due entirely to diet. Further, the food may be good but its preparation bad; the quantity may be sufficiently scientific and the intervals badly judged. It is clear therefore that this malnutrition may be the result of bad quantity of milk, either a deficient or over great quantity, bad timing of feeding or ignorant methods of food preparation; or a combination of these factors. And the perplexing question is always, which of these causes or combinations has produced this condition, and again if the result of one factor, what particular element in its make up is to be suspected.

Out of 200 babies registered at one of our milk stations, 17 we classed as suffering from malnutrition, 11 of these being bottle fed and six breast fed. This includes only those cases due to dietary causes. No special food was at fault, condensed milk and Horlick's however were the worst offenders. Mellin's and Eskay's were noticeably absent, due to the fact that these are generally used with cow's milk. Cow's milk had been tried in most cases but the method and strength had been out of all proportion to the digestibility of the child; consequently the food was unsuccessful. We cannot emphasize this too strongly. Where there is a failure charged to cow's milk, the suspicion must be strong that its strength was at fault.

Heretofore we considered that the top milk method was the last word in the feeding of babies, but this is now a thing of the past and only used in special cases. Today we are convinced that simplicity is the key note; therefore the reason for giving the proprietary foods, that their preparation was easy, is no longer a justification for either use. We must admit, however, that there are many cases fed successfully on the proprietary mixtures and at times are valuable as a temporary food, but I have yet to see a dissatisfied mother after her babe has been changed to cow's milk.

There are three gradations of this condition of dietary malnutrition which I will divide clinically for the sake of clearness. First class, mild malnutrition in the breast fed. The weight is about two pounds under the average; the muscles are somewhat flabby; there is mild anemia; the child is restless and irritable. It usually occurs at three periods; at two months of age when the mother has tried to nurse and is unsuccessful, at six months of age when the breast supply naturally begins to diminish in quality and quantity and at the 14th or 15th month when the nursing is prolonged. Many of these cases develop the early symptoms of rickets, and we should constantly be on the watch. In 15 cases of rickets entered at one of our milk stations 7 were in the breast fed, due to nervous stations well predominating. The early stages of rickets in all of these cases occurred within the above periods. Therefore, it is important that we should keep this fact in mind.

Example—of breast fed, malnutrition. Chas Bell, weight at birth 7 pounds and 8 ounces. Admitted to the milk station at ten weeks of age, weight 7 pounds and 14 ounces. Mother complained that the child cried constantly, was never satisfied and wanted to nurse continually. The stools were green and there was occasionally vomiting. We gave cow's milk, starting with one ounce of milk and 3 ounces of water, with the addition of one-half teaspoonful cane sugar. In two weeks the food was increased to two and one-half of milk and the same of water, with one teaspoonful sugar to each feeding. The vomiting ceased, the stools became better and we had a contented baby. At the present time the baby has gained on an average of 8 ounces each week. If this mother had given a proprietary food the result might have been disastrous. These cases tolerate milk well because there has been no great damage to the digestive system.

The second class is the moderate degree of malnutrition in the bottle fed. These babies have been fed on one or two foods for a considerable length of time, usually months. The first food not producing a satisfactory weight another is tried with the same result. The foods are ordinarily well digested and the child is fairly comfortable, yet the weight is stationary or the gain is very slight. The addition of cow's milk to the dietary of these cases is all that is necessary to get the desired result. But we must again urge the caution that all amounts be at the beginning conservative and increased as the toleration indicates. A baby moderately hungry for a day or two is far better off than one almost killed by kindness.

The third class is characterized by a constant loss of weight, anemia, flabby muscles, vomiting or diarrhoea and constant crying. Many of these cases are either luetic or tubercular but give no definite symptoms or positive reactions. These are the cases more annoying and vexing to the physician than many a contagious disease. Some do not thrive on any food but become anaemic.

Example—Baby Ford, admitted to the milk station at two months of age, weight 5 pounds and 6 ounces. The weight at birth was seven pounds. All kinds of foods had been tried.

Cow's milk was given at one month of age, half milk and half water, six ounces in all. We could not expect this strength and quality to be successful considering the digestibility of the child. It vomited the milk and had green stools, so another food was tried. There was no clinical evidence of lues, Wassermann was refused, VonPirquet was negative. Scurvy was suggested because of the apparent pain in handling the baby, especially when the diapers were changed. The treatment consisted in giving skim milk, boiled and peptonized. Later the cream was added and the peptonizing was omitted. Cane sugar was used in one-half teaspoonful to each bottle. Curds would appear in the stools and the baby showed symptoms of being distressed. The cream was removed and there was a cessation of the symptoms. When the cane sugar was increased the stools became more frequent. Dextro-maltose was given in place of cane sugar but was immediately vomited. 2% cane sugar solution was then added for the regular diluent. The cereal gruels either plain or dextrinized were not tolerated, water being the most satisfactory diluent. It must be remembered, however, that in many of these cases the gruels are of great benefit and should always be tried in a slow growing infant. One teaspoonful of beef juice was given in every bottle and one teaspoonful orange juice given three times daily. This baby weighs now 12 and one-half pounds, a gain of one pound a month, in spite of its many upsets and diet of skim milk which was given probably half of the time. It evidently had an intolerance to fats as well as sugars. This is a type of case which is far too common and the treatment is discouraging but with a careful study of the child's tolerance, the final outcome is usually successful. I could cite many other cases of this class where the gain was more rapid and the treatment more satisfactory.

The treatment of malnutrition is dietary and general. Breast feeding should always be encouraged and every effort made to maintain it where there is normal growth. When the breast is not satisfactory, one should not be too conservative in putting infants on the bottle. When breast milk is good, it is better than good artificial feeding, but good artificial feeding is always better than poor breast milk. We now believe that longer intervals in the breast fed are conducive to better health. Most breast cases do better on 3 or 4 hour intervals when previously we advised 2 or 3 hour intervals. I am not satisfied about the efficacy of these intervals in the bottle fed. When commencing on cow's milk, it is well to give a cathartic, a bowel wash and a 12 hour hunger diet of saccharin tea solution. We have recognized recently that too long a starvation period is actually injurious to the child. In the mild cases it may only be necessary to add cow's milk gradually and at the same time to withdraw the present food. The breast cases are handled the same way, unless there are reasons for sudden weaning.

In regard to the modification of cow's milk in these cases, a very weak mixture is essential at first. Then the mixture is gradually increased according to the digestion of the child. In infants of normal digestion and those of the malnutrition type where the digestion is not seriously impaired, the ordinary milk formula is satisfactory. This is briefly as follows:

| | | | | |
|---|---|---|---|---|
| First mo. | 1 oz. Milk | 2 oz. 5% sugar water | 2 hr. intv. | 10 feed |
| 2nd | 1½ | 2½ | 2½ | 8 |
| 3 and 4 | 2½ | 2½ | 3 | 7 |
| 5th | 3½ | 2½ | 3 | 6-7 |
| 6 | 4 | 2 | 3 | 6 |
| 7 | 5 | 2 | 3 | 5 |
| 8-10 | 6 | 2 | 3 | 4 |

You will notice that we have made no mention of the percentages and properly so because they are somewhat complicating and unnecessary. They can be figured out for reference. This method of feeding as well as other methods is often attended with some food injury. Our greatest error in this connection has been the erroneous idea concerning the curdy stool. This curd according to Finklestein is not undigested casein but composed of fatty acids and bacteria, and is only seen in babies fed on raw milk. Therefore, the fats and sugars are the usual disturbing elements in milk. Proteids are digested in relatively high percentages without any disturbance. This is shown by the use of albumen milk in cases of weak digestion. The treatment of a fat injury therefore is not necessarily the withdrawal of the cream but a change or diminished sugar content. The stool resulting from a sugar injury is apt to be liquid, foul odor, sometimes green, with varying amounts of mucus, the treatment of which is a withdrawal of the sugar or a change in the kind of sugar. Are there infants who will not tolerate cow's milk? Occasionally, we see a case where the milk is digested with great difficulty but an actual idiosyncrasy is rare.

Where the digestion is very feeble or has been badly damaged as in the severe cases of malnutrition there are various ways to adapt cow's milk to the digestion. Skim milk boiled will answer in the majority of cases. Boiling the milk increases the digestibility.

Buttermilk is practically the same as skim milk but with an increased amount of lactic acid. This is advantageous in certain cases of weak digestion. It may easily be made from the lactone tablet. Buttermilk with the addition of a gruel and sugar may be used for a long time with good results.

The use of whey we have found beneficial in some cases notwithstanding the fact that the sugar and salt content is injurious to weak digestion. Babies fed on whey are apt to have flatulence. The stools do not improve in number but rather in quality. Skim milk is usually added to the whey when there is an indication for more food.

Albumen milk is especially useful when the digestion is intolerant to all other foods, but on account of the difficulty of preparation it is not practicable in the majority of homes. It can be made, however, if enough attention is paid to details. I would recommend a more general use of this milk in all disturbances of digestion.

In the severe disturbances of digestion, either of the enteritis type or the intoxication type, the question of food is a very important one. The malnutrition which follows this condition is very apt to be severe because so much weight has been lost either through fluid stools or the using up of the body energy by toxemia. Whether this loss has been acute or protracted the digestive resistance has been greatly lowered so that the feeding at this time must be a careful procedure.

It is the usual method and rightly so to begin after a hunger diet with the former food in small amounts. This will be tolerated in the mild cases but not in the severe cases. If a child has been taking cow's milk before a disturbance the chances are that it will not digest it at this time, but we may start on some proprietary milk with a moderate degree of safety. If, however, the child has had a proprietary mixture before an intestinal disturbance then the problem is more difficult.

It is in this type of cases that I think Eiweiss milk is especially indicated. As a usual thing the child will not gain on this milk and it is not given with that intention, but the general condition will improve. Later, cow's milk may be added gradually according to the tolerance of the digestion.

Next in importance to diet is general management, air being the most essential. Many of these patients will not improve on any formula until fresh air is secured. (Ginsberg). The natural tendency of a mother who has a delicate infant is to keep it closely confined for fear of its catching cold. In the country you do not have this problem so manifest. Cool sponging, massage, cod liver oil, nux vomica, beef juice and orange juice, all help in the general care.

In conclusion let me say that no matter what kind or degree of malnutrition, cow's milk should be the ultimate aim in feeding. This method which I have given you commends itself for its simplicity. Feedings are easy to prepare and it is successful with the ordinary grade of cow's milk. I must emphasize, however, the necessity of a close scrutiny in every case, the several factors that may produce infantile malnutrition. A conservative beginning with the whole milk method and a gradual increase as fast as tolerance can be ascertained. With a fair knowledge in the use of skim milk, whey, albumen milk and a limited use of the proprietary foods the ordinary cases of malnutrition can be treated successfully.

572 W. Ferry St., Buffalo, N. Y.

---

**Treatment of War Wounds in the Period of Attack.** Marquis, Descazals, Luquet and Morlot, describe the method of securing prompt definitive treatment. A large operative pavilion is established as near the front as possible, with Y operating rooms and the necessary annexes, carried by 11 surgical and radiologic trucks. The wounded receive attention in 6-12 hours from the time of the wound. Congestion is prevented by providing special ambulances for the moribund and the slightly wounded, 3 kilometers in advance of the hospital. By eliminating the process of sorting and attending to these cases, 178 patients have been cared for at the mobile operating hospital in 24 hours. They present the following statistics: 298 wounded represented 550 different wounds. Of these, 342 were of soft parts, 133 osseous, 34 articular, 15 cranial including 11 with the meninges opened, 19 of the abdomen including 18 visceral, 12 of the thorax, of which 5 involved the lung and 7 only the pleura, 109 wounds of all kinds were sutured primarily, 370 secondarily—total closures 469, 85% of all the series.

59 days after the attack, 108 of the 298 wounded, 36%, had been discharged convalescent. 109, 36% were practically healed and awaiting discharge, some on leave, some to centers of further treatment, 13, 4%, had been evacuated healed, to centers of mechanotherapy, neurology, etc., for muscular training, etc. 45, 15%, had not been benefitted by suture, the majority of them will be healed without further attempt. 21 died after operation, 9 before, a total mortality in the series, of 6.5%.

---

**Venereal Disease Incidence in the Army.** The aggregate incidence, Sept.-Dec., 1917, was 162.4 per mille for the N. A, 115.2 for the N. G. and 88 for the regular army. The first two proportions apply to conditions at time of enlistment. The value of discipline and instruction and the fact that venereal disease is a civil and not a military danger, are clearly demonstrated.

